# Imaging of Subsurface Corrosion Using Gradient-Field Pulsed Eddy Current Probes with Uniform Field Excitation

**DOI:** 10.3390/s17081747

**Published:** 2017-07-31

**Authors:** Yong Li, Shuting Ren, Bei Yan, Ilham Mukriz Zainal Abidin, Yi Wang

**Affiliations:** 1 State Key Laboratory for Strength and Vibration of Mechanical Structures, Shaanxi Engineering Research Center of NDT and Structural Integrity Evaluation, Xi’an Jiaotong University, Xi’an 710049, China; renshuting1@stu.xjtu.edu.cn (S.R.); yanbei@stu.xjtu.edu.cn (B.Y.); wybit2008@stu.xjtu.edu.cn (Y.W.); 2 Leading Edge NDT Technology (LENDT) Group, Malaysian Nuclear Agency, Bangi 43000, Kajang, Selangor, Malaysia; mukriz@nuclearmalaysia.gov.my

**Keywords:** electromagnetic nondestructive evaluation, gradient-field pulsed eddy current inspection, subsurface corrosion, analytical modeling, corrosion imaging, uniform field excitation

## Abstract

A corrosive environment leaves in-service conductive structures prone to subsurface corrosion which poses a severe threat to the structural integrity. It is indispensable to detect and quantitatively evaluate subsurface corrosion via non-destructive evaluation techniques. Although the gradient-field pulsed eddy current technique (GPEC) has been found to be superior in the evaluation of corrosion in conductors, it suffers from a technical drawback resulting from the non-uniform field excited by the conventional pancake coil. In light of this, a new GPEC probe with uniform field excitation for the imaging of subsurface corrosion is proposed in this paper. The excited uniform field makes the GPEC signal correspond only to the field perturbation due to the presence of subsurface corrosion, which benefits the corrosion profiling and sizing. A 3D analytical model of GPEC is established to analyze the characteristics of the uniform field induced within a conductor. Following this, experiments regarding the imaging of subsurface corrosion via GPEC have been carried out. It has been found from the results that the proposed GPEC probe with uniform field excitation not only applies to the imaging of subsurface corrosion in conductive structures, but provides high-sensitivity imaging results regarding the corrosion profile and opening size.

## 1. Introduction

Conductive structures of nonmagnetic materials such as aluminum and copper are widely employed in engineering fields involving energy, transportation, as well as aerospace. Despite anti-corrosion measures, in-service conductive structures are still vulnerable to corrosion due to hostile and particularly corrosive environments [1,2]. Among various types of corrosion, subsurface corrosion poses the most severe threat to structural integrity. The reason lies in the fact that it normally occurs either within the conductor body or on the back surface of the conductor. Such conventional non-destructive evaluation (NDE) techniques as visual testing (VT) [3], penetrant testing (PT) [4], single-frequency eddy current testing (ECT) [5] and magnetic particle inspection (MPI) [6], etc., which target cracks in industry may leave subsurface corrosion undetected. Therefore, it is highly desirable to noninvasively evaluate and in particular visualize subsurface corrosion in conductive structures via appropriate NDE methods before catastrophic accidents happen.

**Figure 1:**
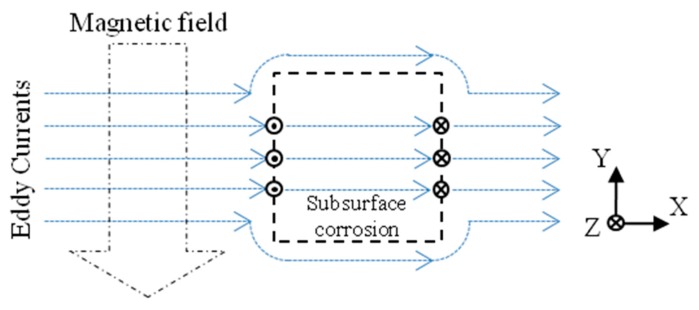
Schematic illustration regarding the interaction of the uniform eddy current with subsurface corrosion (top view).

Gradient-field pulsed eddy current technique (GPEC) is an extension of the pulsed eddy current technique (PEC), which is capable of evaluating the integrity of a conductive structure with a thickness up to 10 mm [7]. It has been found to be superior in the high-sensitivity evaluation of hidden material degradation and corrosion in conductors [8,9]. Even though subsurface corrosion could be visualized using GPEC probes, which consist of pancake coils for the excitation of the incident/primary magnetic field and magnetic sensors for quantifying the gradient of the magnetic field (namely the gradient field), there is a large discrepancy between the true corrosion profile and imaging result [8]. It could be mostly because of the fact that the incident magnetic field excited by pancake coils is non-uniform and thus, the acquired signals from magnetic sensors detect the gradient field resulting from not only the distortion of eddy currents due to anomalies in the conductor under inspection, but also the original incident magnetic field. This technical drawback arising from the previous probe configuration opens up the optimization of GPEC probes by realizing uniform field excitation, which gives localized uniform distributions of the eddy current and incident magnetic field. A schematic illustration exhibiting the interaction of the uniform eddy current induced in a conductor with subsurface corrosion is presented in Figure 1. It can be noticed from Figure 1 that in corrosion-free region the gradient field is null due to uniform distributions of the eddy currents and the incident magnetic field. In contrast, in the defect area and particularly at the edges of the subsurface corrosion, the eddy current is significantly disturbed due to material discontinuity, thus leading to the gradient field. In such case, the gradient-field signal is independent of the incident magnetic field, but only relies on the presence of subsurface corrosion. In view of this, a GPEC probe with uniform field excitation could be beneficial to high-sensitivity imaging for the profiling and sizing of subsurface corrosion.

As the key technology, uniform field excitation plays a vital role in alternating current field measurement (ACFM), which uses the sinusoidal excitation current, and is usually used for the detection and sizing of cracks in conductive structures [10]. In order to perform crack sizing, LeTessier et al. adopted an induction coil with its lateral surface facing the conductor surface to induce uniform eddy currents in the conductive areas of contact heaters and tanks [11]. By using a probe with the similar configuration, Knight et al. intensively investigated the influence of residual stress on ACFM for the inspection of cracks in drill-collar threaded connections [12]. Li et al. used an encircling coil for the generation of a uniform field in the external surfaces of pipes for the detection of cracks [13]. For arbitrary-angle cracks in planar conductors, Li et al. also proposed a double U-shaped orthogonal inducer to induce the uniform eddy current whose main axis could rotate at each scanning point [14]. It is believed that along with uniform field excitation, GPEC responses to subsurface corrosion, especially to its profile, could be enhanced. However, to the authors’ knowledge the application of uniform field excitation to GPEC in the detection and imaging of subsurface corrosion has barely been investigated.

In this paper, a new GPEC probe together with uniform field excitation is proposed for the profiling and sizing of subsurface corrosion in nonmagnetic planar conductors through corrosion imaging. The uniformities of the eddy current and magnetic field are investigated through simulations based on their closed-form expressions formulated via the analytical modeling i.e., extended truncated region eigenfunction expansion (ETREE) modeling [15]. Following this, experiments were carried out in order to assess the capability of the proposed GPEC probe in high-sensitivity imaging of subsurface corrosion in conductors. The imaging accuracy in terms of the profile identification and estimation of the opening size of subsurface corrosion is evaluated.

## 2. Field Formulation and Investigation of Uniform Field Characteristics

### 2.1. Field Formulation

**Figure 2:**
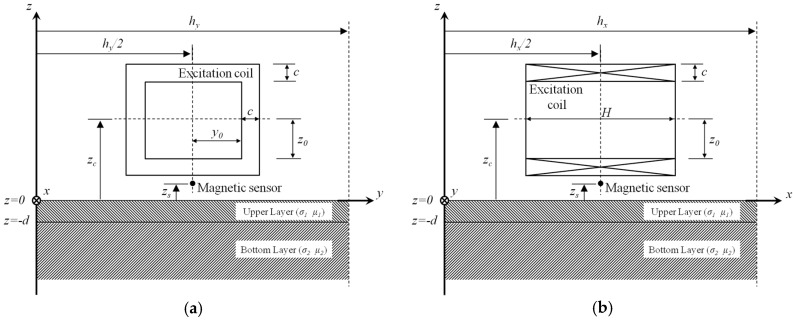
Model of the proposed GPEC probe placed over a two-layer conductor. (**a**) Side view in *x*-direction; (**b**) side view in *y*-direction.

In difference to the previous probe configuration [8,9], the proposed GPEC probe consists of: (1) a rectangular coil (in lieu of the pancake coil) for generating the incident magnetic field; and (2) a magnetic sensor for measuring the gradient field. During inspection, it is placed over the upper surface of a layered conductor. It is noteworthy that in a bid to implement uniform field excitation, the rectangular coil is perpendicularly placed on the conductor with its lateral winding facing the conductor’s upper surface. The model is shown in Figure 2.

Based on ETREE modeling [15,16,17,18], the net magnetic field in the air gap between the rectangular coil and conductor is written as:

(1) $$\left\{ \begin{array}{l} \vec{B}\left(t\right)=\frac{16\mu_{0}NI\left(t\right)}{Hch_{x}h_{y}}⊗\sum_{m=1}^{\infty }\sum_{n=1}^{\infty }C_{mn}\left\{\left[e^{\kappa_{mn}z}-e^{-\kappa_{mn}z}\zeta_{mn}\left(t\right)\right]\left(u_{m}\eta_{1}{\vec{x}}_{0}+v_{n}\eta_{2}{\vec{y}}_{0}\right)+\left[e^{\kappa_{mn}z}+e^{-\kappa_{mn}z}\zeta_{mn}\left(t\right)\right]\kappa_{mn}\eta_{3}{\vec{z}}_{0}\right\}\\ \eta_{1}=\cos\left(u_{m}x\right)\sin\left(v_{n}y\right);\;\eta_{2}=\sin\left(u_{m}x\right)\cos\left(v_{n}y\right);\;\eta_{3}=\sin\left(u_{m}x\right)\sin\left(v_{n}y\right) \end{array}\right.$$

Following Equation (1), the closed-form expression of the signal regarding each component of the gradient field $g\left(\vec{B}\right)$, $g\left(\vec{B}\right)=\nabla \vec{B}$ measured by the magnetic sensor at an arbitrary location (*x*, *y*, *z_s_*) between the rectangular coil and conductor is formulated as:

(2) $$\left\{ \begin{array}{l} g_{x}\left(\vec{B}\right)=\frac{16\mu_{0}NI\left(t\right)}{Hch_{x}h_{y}}⊗\sum_{m=1}^{\infty }\sum_{n=1}^{\infty }u_{m}C_{mn}\left\{\left[e^{-\kappa_{mn}z_{s}}\zeta_{mn}\left(t\right)-e^{\kappa_{mn}z_{s}}\right]\left(u_{m}\eta_{1x}{\vec{x}}_{0}-v_{n}\eta_{2x}{\vec{y}}_{0}\right)+\left[e^{\kappa_{mn}z_{s}}+e^{-\kappa_{mn}z_{s}}\zeta_{mn}\left(t\right)\right]\kappa_{mn}\eta_{3x}{\vec{z}}_{0}\right\}\\ g_{y}\left(\vec{B}\right)=\frac{16\mu_{0}NI\left(t\right)}{Hch_{x}h_{y}}⊗\sum_{m=1}^{\infty }\sum_{n=1}^{\infty }v_{n}C_{mn}\left\{\left[e^{\kappa_{mn}z_{s}}-e^{-\kappa_{mn}z_{s}}\zeta_{mn}\left(t\right)\right]\left(u_{m}\eta_{1y}{\vec{x}}_{0}-v_{n}\eta_{2y}{\vec{y}}_{0}\right)+\left[e^{\kappa_{mn}z_{s}}+e^{-\kappa_{mn}z_{s}}\zeta_{mn}\left(t\right)\right]\kappa_{mn}\eta_{3y}{\vec{z}}_{0}\right\}\\ g_{z}\left(\vec{B}\right)=\frac{16\mu_{0}NI\left(t\right)}{Hch_{x}h_{y}}⊗\sum_{m=1}^{\infty }\sum_{n=1}^{\infty }\kappa_{mn}C_{mn}\left\{\left[e^{\kappa_{mn}z_{s}}+e^{-\kappa_{mn}z_{s}}\zeta_{mn}\left(t\right)\right]\left(u_{m}\eta_{1}{\vec{x}}_{0}+v_{n}\eta_{2}{\vec{y}}_{0}\right)+\left[e^{\kappa_{mn}z_{s}}-e^{-\kappa_{mn}z_{s}}\zeta_{mn}\left(t\right)\right]\kappa_{mn}\eta_{3}{\vec{z}}_{0}\right\} \end{array}\right.$$

where,

(3) $$\left\{ \begin{array}{l} \eta_{1x}=\sin\left(u_{m}x\right)\sin\left(v_{n}y\right);\;\eta_{2x}=\cos\left(u_{m}x\right)\cos\left(v_{n}y\right);\;\eta_{3x}=\cos\left(u_{m}x\right)\sin\left(v_{n}y\right)\\ \eta_{1y}=\cos\left(u_{m}x\right)\cos\left(v_{n}y\right);\;\eta_{2y}=\sin\left(u_{m}x\right)\sin\left(v_{n}y\right);\;\eta_{3y}=\sin\left(u_{m}x\right)\cos\left(v_{n}y\right) \end{array}\right.$$

In Equations (1) and (2), ⊗ denotes convolution. ${\vec{x}}_{0}$, ${\vec{y}}_{0}$ and ${\vec{z}}_{0}$ are unit vectors. *μ*_0_ is the permeability of vacuum. *I*(*t*) and *N* stand for the excitation current in an arbitrary waveform and the number of turns of the rectangular coil, respectively. $\kappa_{mn}=\sqrt{u_{m}^{2}+v_{n}^{2}}$, where, *u_m_* = *m*π/*h_x_* and *v_n_* = *n*π/*h_y_* (*m* and *n* are integers). The other terms include [19,20]:

(4) $$\left\{ \begin{array}{l} C_{mn}=\frac{\Phi_{mn}\cos\left(u_{m}h_{x}/2\right)\sin\left(u_{m}H/2\right)\sin\left(v_{n}h_{y}/2\right)e^{-\kappa_{mn}z_{c}}}{\kappa_{mn}^{2}v_{n}}\\ \Phi_{mn}=\frac{1}{\kappa_{mn}^{2}+v_{n}^{2}}\left\{\begin{array}{l} \kappa_{mn}\sin\left[v_{n}\left(y_{0}+c\right)\right]\cosh\left[\kappa_{mn}\left(z_{0}+c\right)\right]-\kappa_{mn}\sin\left(v_{n}y_{0}\right)\cosh\left(\kappa_{mn}z_{0}\right)\\ -v_{n}\cos\left[v_{n}\left(y_{0}+c\right)\right]\sinh\left[\kappa_{mn}\left(z_{0}+c\right)\right]+v_{n}\cos\left(v_{n}y_{0}\right)\sinh\left(\kappa_{mn}z_{0}\right) \end{array}\right\} \end{array}\right.$$

It is noteworthy that in Equations (1) and (2) *ζ_mn_*(*t*) denotes the time-domain expression of the conductor reflection coefficient [8,21]. It can be readily computed via inverse Fourier transform of its time-harmonic form *ζ_mn_*(*ω*), where, *ω* denotes the angular frequency of each harmonic in the spectrum of the excitation current [22]. For a two-layer conductor comprising an upper layer with the finite thickness *d* and a bottom layer with infinite thickness (as shown in Figure 2), *ζ_mn_*(*ω*) can be written as:

(5) $$\left\{ \begin{array}{l} \zeta_{mn}\left(\omega \right)=\frac{1}{\rho_{mn}}\left[\left(\lambda_{1}\mu_{2}+\lambda_{2}\mu_{1}\right)\left(\kappa_{mn}\mu_{1}-\lambda_{1}\right)+e^{-2\lambda_{1}d}\left(\lambda_{1}\mu_{2}-\lambda_{2}\mu_{1}\right)\left(\kappa_{mn}\mu_{1}+\lambda_{1}\right)\right]\\ \rho_{mn}=\left(\lambda_{1}\mu_{2}+\lambda_{2}\mu_{1}\right)\left(\kappa_{mn}\mu_{1}+\lambda_{1}\right)+e^{-2\lambda_{1}d}\left(\lambda_{1}\mu_{2}-\lambda_{2}\mu_{1}\right)\left(\kappa_{mn}\mu_{1}-\lambda_{1}\right) \end{array}\right.$$

where, $\lambda_{i}=\sqrt{\kappa_{mn}^{2}+j\omega \sigma_{i}\mu_{i}\mu_{0}}$, *i* = 1, 2. *σ_i_* and *μ_i_* denote the conductivity and relative permeability of each layer, respectively.

Following Equation (1), the density of eddy currents induced at an arbitrary position within the upper layer is formulated as:

(6) $${\vec{J}}_{ec}\left(t\right)=\frac{16\mu_{0}\sigma_{1}N\left\{\partial \left[I\left(t\right)\right]/\partial t\right\}}{Hch_{x}h_{y}}⊗\sum_{m=1}^{\infty }\sum_{n=1}^{\infty }C_{mn}\left(-v_{n}\eta_{2}{\vec{x}}_{0}+u_{m}\eta_{1}{\vec{y}}_{0}\right)\left[e^{\kappa_{mn}z}\alpha_{mn}\left(t\right)+e^{-\kappa_{mn}z}\beta_{mn}\left(t\right)\right]$$

It is noted that due to the characteristics of eddy currents in flawless conductors, the *z*-component of ${\vec{J}}_{ec}$ vanishes [23]. In Equation (6), *α_mn_*(*t*) and *β_mn_*(*t*) can be readily recovered through the inverse Fourier transform of their time-harmonic forms *α_mn_*(*ω*) and *β_mn_*(*ω*), respectively. *α_mn_*(*ω*) and *β_mn_*(*ω*) are written as:

(7) $$\left\{ \begin{array}{l} \alpha_{mn}\left(\omega \right)=\left[2\mu_{1}\kappa_{mn}\left(\lambda_{1}\mu_{2}+\lambda_{2}\mu_{1}\right)\right]/\rho_{mn}\\ \beta_{mn}\left(\omega \right)=\left[2\mu_{1}\kappa_{mn}\left(\lambda_{1}\mu_{2}-\lambda_{2}\mu_{1}\right)e^{-2\lambda_{1}d}\right]/\rho_{mn} \end{array}\right.$$

Considering a conductive plate under inspection, *σ*_2_ = 0 MS/m and *μ*_2_ = 1. Therefore, Equations (5) and (7) can further be simplified into:

(8) $$\left\{ \begin{array}{l} \zeta_{mn}\left(\omega \right)=\left\{\left[{\left(\kappa_{mn}\mu_{1}\right)}^{2}-\lambda_{1}^{2}\right]\left(1-e^{-2\lambda_{1}d}\right)\right\}/\rho_{mn}\\ \rho_{mn}={\left(\lambda_{1}+\kappa_{mn}\mu_{1}\right)}^{2}-e^{-2\lambda_{1}d}{\left(\lambda_{1}-\kappa_{mn}\mu_{1}\right)}^{2} \end{array}\right.$$

(9) $$\left\{ \begin{array}{l} \alpha_{mn}\left(\omega \right)=\left[2\mu_{1}\kappa_{mn}\left(\lambda_{1}+\kappa_{mn}\mu_{1}\right)\right]/\rho_{mn}\\ \beta_{mn}\left(\omega \right)=\left[2\mu_{1}\kappa_{mn}\left(\lambda_{1}-\kappa_{mn}\mu_{1}\right)e^{-2\lambda_{1}d}\right]/\rho_{mn} \end{array}\right.$$

It should be pointed out that since GPEC normally utilizes the excitation current in quasi-rectangular waveform, *I*(*t*) in Equations (1), (2) and (6) can thus be analytically formulated in the form of a Fourier series as:

(10) $$I\left(t\right)=I_{0}\left\{\left[\upsilon +\frac{1}{T}\tau e^{-\frac{\upsilon T}{\tau }}\left(1-e^{\frac{T}{\tau }\left(2\upsilon -1\right)}\right)\right]+\frac{1}{\pi }\sum_{l=1}^{\infty }\left[a_{l}\cos\left(\frac{2l\pi t}{T}\right)+b_{l}\sin\left(\frac{2l\pi t}{T}\right)\right]\right\}$$

where *I*_0_, *T*, *υ* and *τ* are the maximum amplitude, period, duty cycle and rising/falling time constant of the current signal, respectively. *a_l_* and *b_l_* are written as:

(11) $$a_{l}=\frac{\sin\left(2l\pi \upsilon \right)}{l}-\frac{2\pi }{T}{\left[\frac{1}{\tau^{2}}+{\left(\frac{2l\pi }{T}\right)}^{2}\right]}^{-1}\left\{\frac{1}{\tau }\left[1+e^{\frac{T\left(\upsilon -1\right)}{\tau }}\right]+\left[\frac{2l\pi \sin\left(2l\pi \upsilon \right)}{T}-\frac{\cos\left(2l\pi \upsilon \right)}{\tau }\right]\left(1+e^{-\frac{\upsilon T}{\tau }}\right)\right\}$$

(12) $$b_{l}=\frac{1-\cos\left(2l\pi \upsilon \right)}{l}-\frac{2\pi }{T}{\left[\frac{1}{\tau^{2}}+{\left(\frac{2l\pi }{T}\right)}^{2}\right]}^{-1}\left\{\frac{2l\pi }{T}\left[1+e^{\frac{T\left(\upsilon -1\right)}{\tau }}\right]-\left[\frac{2l\pi \cos\left(2l\pi \upsilon \right)}{T}+\frac{\sin\left(2l\pi \upsilon \right)}{\tau }\right]\left(1+e^{-\frac{\upsilon T}{\tau }}\right)\right\}$$

Equations (1) and (6) facilitate the computation of the electromagnetic field excited by the rectangular coil and subsequent analysis of its characteristics involving the uniformities of: (1) magnetic field over the upper surface of the conductor; and (2) eddy currents within the conductor.

### 2.2. Characteristics of the Uniform Field

Since the uniform field is of great importance for the proposed GPEC probe, it is essential to investigate the characteristics of the excited electromagnetic field and identify the area where the effective uniform field distributes. Simulations based on Equations (1) and (6) are consequently carried out to analyze the field characteristics with the proposed probe whose configuration is exhibited in Figure 2. It is assumed that subsurface corrosion occurs in the back surface of a conductive plate. Therefore, the uniformity of the eddy current in the plate back surface is intensively analyzed whilst the net magnetic field over the plate upper surface is computed in an effort to investigate its uniformity.

In light of the fact that the excitation current in the quasi-rectangular waveform is employed to drive the rectangular coil, special attention is given to the selection of the time instant when the field response is picked up by a magnetic sensor deployed at the location (*h_x_*/2, *h_y_*/2, *z_s_*), to the subsurface corrosion is the highest. It is noted that in such a case, *y*- and *z*-components of the net magnetic field (*B_y_* and *B_z_*) vanish. In a sense of the conductor size, subsurface corrosion with a dimension considerably larger than that of the excitation coil is analogous to the wall-thinning defect. Consequently, in the presence of the subsurface corrosion the plate thickness decreases with Δ*d* from the back surface. By referring to [8,21,22], the response of *x*-component of the net magnetic field *B_x_* to the initial subsurface corrosion with Δ*d*→0 is written as:

(13) $$\underset{\Delta d\to 0}{\lim}\frac{\Delta B_{x}\left(t\right)}{\Delta d}=\frac{\partial \left[B_{x}\left(t\right)\right]}{\partial d}=\frac{16\mu_{0}NI\left(t\right)}{Hch_{x}h_{y}}⊗\sum_{m=1}^{\infty }\sum_{n=1}^{\infty }u_{m}C_{mn}e^{-\kappa_{mn}z}\zeta_{mn}^{\prime }\left(t\right)\cos\left(\frac{m\pi }{2}\right)\sin\left(\frac{n\pi }{2}\right)$$

where, *ζ*^′^*_mn_* (*t*) can be readily recovered via the inverse Fourier transform of ∂[*ζ_mn_*(*ω*)]/∂*d* which is formulated as:

(14) $$\frac{\partial \left[\zeta \left(\omega \right)\right]}{\partial d}=\frac{2\kappa_{mn}\mu_{1}\lambda_{1}^{2}\left[{\left(\kappa_{mn}\mu_{1}\right)}^{2}-\lambda_{1}^{2}\right]}{{\left\{2\kappa_{mn}\mu_{1}\lambda_{1}\cosh\left(\lambda_{1}d\right)+\left[{\left(\kappa_{mn}\mu_{1}\right)}^{2}+\lambda_{1}^{2}\right]\sinh\left(\lambda_{1}d\right)\right\}}^{2}}$$

Equation (13) is subsequently adopted for the computation of the field response to the initial subsurface corrosion in an effort to choose the time instant when the uniformity of the electromagnetic field involving the eddy current and net magnetic field is intensively analyzed.

**Figure 3:**
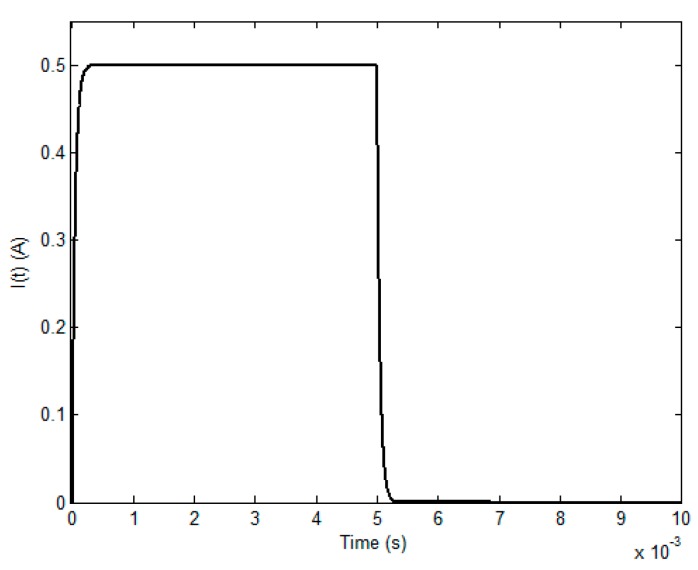
The excitation current *I*(*t*).

**Figure 4:**
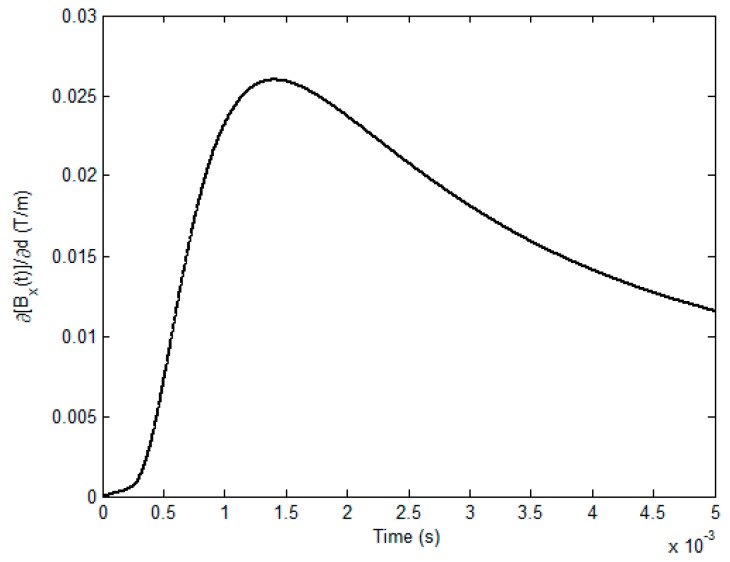
The computed response of *B_x_* to the initial subsurface corrosion (0 ≤ *t* ≤ 5 ms).

Table 1 and Table 2 list the parameters employed in simulations. The material of the conductive plate is Aluminum. As illustrated in Figure 3, the fundamental frequency, duty cycle, rising time and maximum amplitude of the excitation current *I*(*t*) are 100 Hz, 50%, 50 μs and 0.5 A, respectively. By applying Equation (13), the field response to the initial subsurface corrosion is calculated and presented in Figure 4.

**Figure 5:**
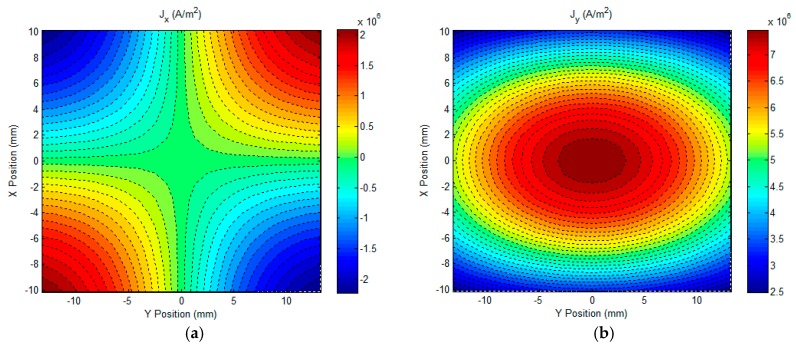
The computed density of eddy currents over the plate back surface. (**a**) *x*-component of the eddy current density *J_x_*; (**b**) *y*-component of the eddy current density *J_y_*.

**Figure 6:**
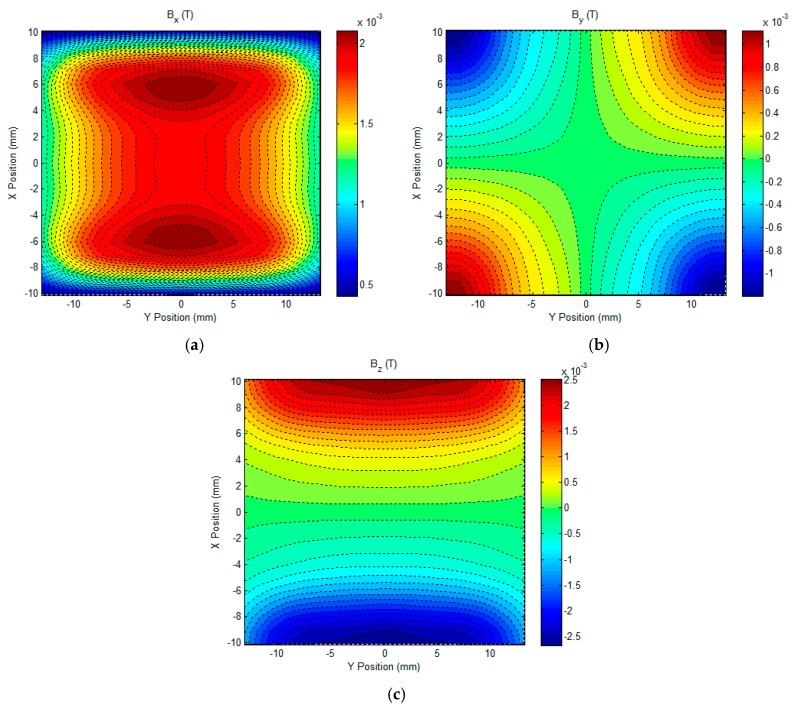
The computed net magnetic field above the plate upper surface. (**a**) *B_x_*; (**b**) *B_y_*; (**c**) *B_z_*.

It can be seen from Figure 4 that the field response to the initial subsurface corrosion is the highest at the time of approximately 1.4 ms. This indicates that due to the presence of the initial subsurface corrosion the perturbation of the eddy current, especially over the back surface of the conductive plate, reaches the maximum at the same time. Further analysis provides the precise temporal value, which is 1406.7 μs, and is used for analysis regarding the uniformities of the eddy current and magnetic field. The calculated distrubtion of the eddy current over the plate back surface and the magentic field above the plate upper surface are exhibited in Figure 5 and Figure 6, respectively. It is noted that the eddy current and magnetic field are invesitigated within the XY plane which covers the lateral surface of the excitation coil (facing the plate upper surface). The coordinate of the plane centre is (0, 0), which corresponds to (*h_x_*/2, *h_y_*/2) in Figure 2.

It can be observed from Figure 5 and Figure 6 that the distributions of the eddy current and net magnetic field in the central region, particularly in the region of interest (ROI) (2 mm × 2 mm area with the center at (0, 0)), where the gradient-field sensor is deployed in experiments are relatively uniform. In a bid to evaluate the uniformity of the uniform field, including the eddy current and net magnetic field within the ROI, an algorithm for the uniformity evaluation regarding the magnetic field [24] is utilized. The computed degrees of field uniformity (DFU) are: 20.6 ppm for the eddy current (averaged value over DFUs of *J_x_* and *J_y_*) and 5.9 ppm for the net magnetic field (averaged value over DFUs of *B_x_*, *B_y_* and *B_z_*), which indicates that in the ROI the eddy current on the plate back surface and the net magnetic field over the plate upper surface are highly uniform. This benefits the high-sensitivity detection and imaging of subsurface corrosion that breaks the field uniformity and thus results in the non-zero gradient-field signal from the GPEC probe.

It is also noticeable from Figure 5 and Figure 6, that in the ROI—compared with the averaged value of *J_x_* which is approximately zero—*J_y_* is over 7 × 10^6^ A/mm^2^, whilst *B_x_* has a considerably larger magnitude (over 1.54 × 10^−3^ Tesla) than *B_y_* and *B_z_*. This implies that: (1) *J_x_*, *B_y_* and *B_z_* are barely sensitive subsurface corrosion on the conductive plate; and (2) the material discontinuity, which is introduced by subsurface corrosion and especially transverse to the direction of *J_y_*, significantly perturbs the distribution of *J_y_*, and thus breaks the uniformity of *J_y_*. As a result, the gradient of the resultant *B_x_* over the plate upper surface, which was originally null for the flawless scenario, is non-zero in the ROI. Consequently, it provides a good implication regarding the presence of subsurface corrosion, and is beneficial to the imaging of subsurface corrosion, particularly its opening profile. In light of this, in the following experimental investigation regarding the GPEC imaging of subsurface corrosion, the *x*-direction gradient of *x*-component of the net magnetic field *g_x_*(*B_x_*) was measured by using a magnetic sensor which is placed right under the lateral winding of a rectangular coil.

## 3. Experiments

### 3.1. System Setup

**Figure 7:**
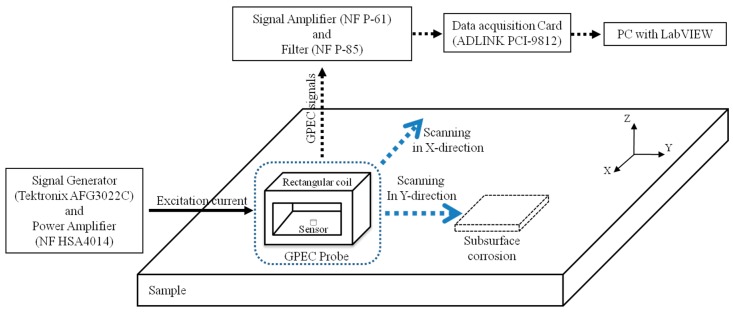
Schematic illustration of the corrosion imaging system with the proposed GPEC probe and sample.

A corrosion imaging system was set up to further assess the applicability of the proposed GPEC probe in the imaging of subsurface corrosion in nonmagnetic planar conductors. The system setup is shown in Figure 7. The parameters of the rectangular coil were the same as those tabulated in Table 1. The pulse repetition frequency and duty cycle of the excitation current driving the rectangular coil were set as 100 Hz and 50%, respectively, whilst it had a maximum amplitude of 0.3 A and a rising time of 58.7 μs, which were directly measured from the acquired current signal. In a bid to acquire signals of the gradient field with high sensitivity, a tunnel magneto-resistance sensor (MultiDimension TMR-4002) was adopted. It was deployed right under the lateral winding of the rectangular coil. It is noted that the component of the net magnetic field and direction of its gradient field, which is sensed by the sensor, were both parallel to the axis of the rectangular coil. For example, for the case shown in Figure 7, the *x*-direction gradient field of the *x*-component of the net magnetic field *g_x_*(*B_x_*) was the main component measured.

A plate of aluminum alloy with subsurface corrosion in different profiles and sizes was fabricated in a bid to simulate the nonmagnetic planar conductor with subsurface corrosion. It was taken as the sample under inspection. The conductivity and thickness of the plate were 33.6 MS/m and 6 mm. It is noted that the plate conductivity was measured via the direct current potential drop method [25,26]. The profile and opening size of each corrosion are tabulated in Table 3.

During experiments, the 2D probe scanning over the plate surface was carried out with a spatial resolution of 0.5 mm. It is noteworthy that in order for the corrosion imaging to be carried out, the 2D probe scanning was conducted twice. After completing the first-round probe scanning with the axis of the rectangular coil parallel to the X axis alongside the signal acquisition of *g_x_*(*B_x_*) at each scanning position, the probe was rotated by 90° in the XY plane, and the second-round probe scanning was carried out along with the measurement of the *y*-direction gradient field of the *y*-component of the net magnetic field *g_y_*(*B_y_*). At each scanning position, particularly within the corrosion region, the peak values (PVs) of the acquired gradient-field signals regarding *g_x_*(*B_x_*) and *g_y_*(*B_y_*)—which are null when the probe is placed over a flawless area of the sample—are extracted in an effort to construct the corrosion images. The final image of every corrosion is derived from the superposition of images regarding *g_x_*(*B_x_*) and *g_y_*(*B_y_*).

### 3.2. Imaging Results and Discussion

**Figure 8:**
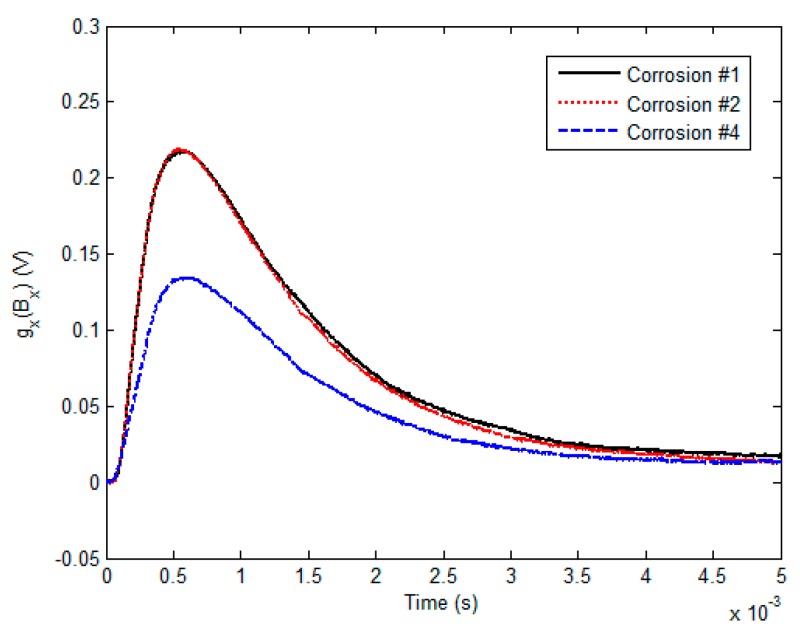
Gradient-field signals with respect to Corrosion #1, Corrosion #2 and Corrosion #4.

Prior to the corrosion imaging, the system was further calibrated by placing the probe above the defect-free area of the sample, and setting the magnitude of the acquired signal as zero. The gradient-field signals from the GPEC probe deployed right over the edges of the subsurface corrosion involving Corrosion #1, Corrosion #2 and Corrosion #4 were firstly investigated, and are exhibited in Figure 8.

It can be seen from Figure 8 that the magnitude of the gradient-field signal changes with the corrosion volume. The PV of the signal rises as the corrosion depth increases, whilst it is insensitive to the corrosion diameter. This indicates that the variation in the gradient-field signal, as well as its PV, mostly relies on the interface thickness at the boundary of a material discontinuity, and could be exploited for approximating the depth of the subsurface corrosion.

**Figure 9:**
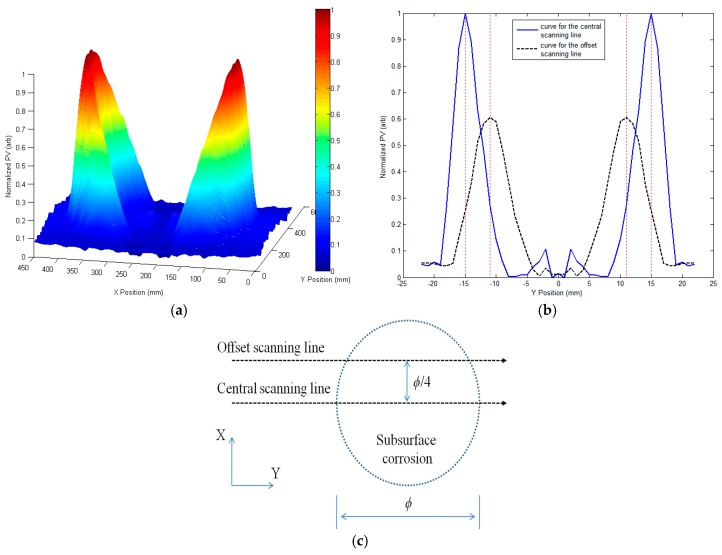
Peak values (PVs) of *g_x_*(*B_x_*) vs. probe positions. (**a**) Scanning curves for Corrosion #1; (**b**) scanning curves along the central and offset scanning lines; (**c**) schematic illustration of the central and offset scanning lines.

Following the probe scanning procedure, multiple scanning curves (PV of the GPEC signal against the probe location) were acquired, and subsequently adopted for deriving the corrosion images. It is noted that apart from Corrosion #6, the coordinate of the corrosion center was set as X = 0, Y = 0. The center of Corrosion #6 was set at the joining point of two circle-shaped regions (C1 and C2 in Table 3). Figure 9 presents the scanning curves (PVs of *g_x_*(*B_x_*) vs. probe positions) when the probe scans over Corrosion #1. The scanning curves along the central and offset scanning lines, which are illustrated in Figure 9c, are shown in Figure 9b along with the locations of the corrosion edges implicated by the dotted lines. Note that the scanning curves are normalized with the maximum amplitude corresponding to 1. It is noticeable from Figure 9 that the corrosion edges can be localized without much loss in accuracy by finding the peaks of the acquired scanning curves. This benefits the subsequent identification of the corrosion profile and the estimation of the corrosion size via GPEC imaging of subsurface corrosion.

**Figure 10:**
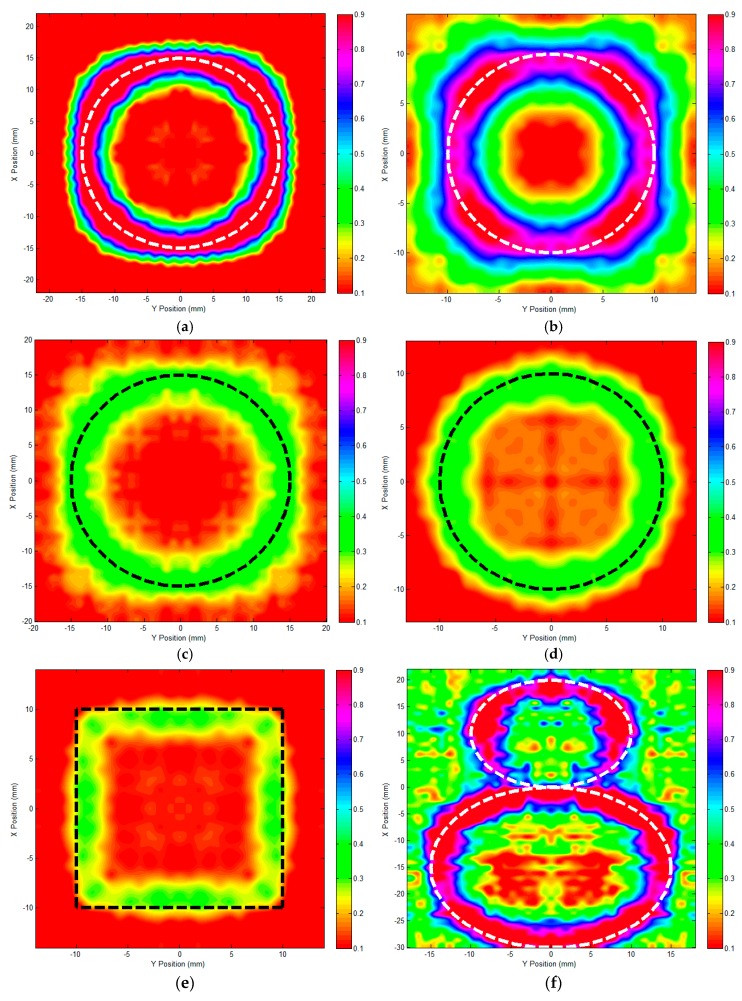
Images of subsurface corrosion via GPEC with uniform field excitation. (**a**) Corrosion #1; (**b**) Corrosion #2; (**c**) Corrosion #3; (**d**) Corrosion #4; (**e**) Corrosion #5; (**f**) Corrosion #6.

After the probe scanning and data processing, the final image for each corrosion was constructed and shown in Figure 10, along with the true corrosion profile indicated by the dashed line. It is noteworthy that all the data i.e., PVs in corrosion images were normalized with the maximum PV corresponding to 1, following which the hue-saturation value (HSV) color was utilized to map the normalized PVs for the production of corrosion images.

It can be qualitatively observed from Figure 10 that: (1) each subsurface corrosion can be detected; and (2) the profile/shape of each subsurface corrosion can be directly visualized and identified by using the proposed GPEC probe with uniform field excitation. The corrosion profile implied by the image is in good agreement with the true profile of each corrosion. This is beneficial to the determination regarding the profile of subsurface corrosion in the conductor. Further analysis has revealed that the difference in HSV components, i.e., image contrast of the corrosion image, is highly dependent on the corrosion depth. This is because the distortion of the eddy currents due to subsurface corrosion—which results in the gradient-field signal—depends mostly on the interface thickness at the boundary of the material discontinuity. The image contrast is enhanced as the corrosion depth increases, whilst the image has lower contrast when the detected corrosion is shallow. This implies that: (1) the image quality for corrosion detection and sizing is dependent on the corrosion dimension, particularly its depth; and (2) the image contrast could be used for further evaluation of the corrosion depth. It is also noticeable from Figure 10f that the corrosion image for Corrosion #6 with the complex profile is seemingly “noisy”. The reasoning could lie in the fact that the gradient field resulting from the small circle-shaped area (C1 in Table 3) interferes with that of the large section (C2 in Table 3), which leads to a more complicated distribution of the gradient field for Corrosion #6 than the other corrosion scenarios. Whereas the profiles of Corrosion #6 and particularly two corrosion sections (C1 and C2) can still be identified. Therefore, by using the proposed GPEC probe with uniform field excitation, multiple subsurface corrosions close to each other could be individually detected together with their profiles visualized by corrosion imaging.

Following the identification of the corrosion profile, the corrosion sizing based on acquired images was further investigated. The maximum magnitude in each image was extracted in an effort to quantitatively assess the diameter/length of each subsurface corrosion. The comparison regarding the corrosion size between the estimated and true values is presented in Table 4. It can be seen from Table 4 that for the given subsurface corrosion the approximated diameter/length of each subsurface corrosion is in good agreement with the true value. The maximum relative error is less than 10%. It is also noticeable that the discrepancy between the estimated and true values increases when either the depth or diameter/length of the corrosion has decreased, which is because of the drop in evaluation sensitivity due to less perturbation of eddy currents at the corrosion boundary. This indicates that the detection and sizing of subsurface corrosion depends on its dimension. Additional image processing techniques should be employed in an effort to enhance the accuracy in sizing of either shallow or small-volume corrosion. Nevertheless, it is noteworthy from Figure 10 and Table 4 that the corrosion imaging based on GPEC with uniform field excitation is promising in not only the identification of the corrosion profile but also the quantitative evaluation of the opening size of subsurface corrosion in nonmagnetic planar conductors without much loss in accuracy.

## 4. Conclusions

In this paper, a GPEC probe with uniform field excitation was proposed for the imaging of subsurface corrosion within nonmagnetic conductors. A 3D analytical model of the proposed GPEC probe was established, and the closed-form expression of the magnetic field, gradient field and eddy current density were formulated. The characteristic uniformities of the electromagnetic field, including the magnetic field above the conductor and the eddy current density on the conductor back surface, were investigated via a series of simulations based on analytical modeling. Following this, experiments regarding the imaging of subsurface corrosion by using the proposed GPEC probe were conducted. It can be found from the experimental results that: (1) the magnitude of the GPEC signal, image contrast and sizing accuracy regarding subsurface corrosion are dependent on the corrosion dimension, especially its depth; and (2) the proposed GPEC probe with uniform field excitation is capable of not only identifying the corrosion profile, but also of providing an estimation regarding the opening size of subsurface corrosion in nonmagnetic planar conductors without much loss in accuracy.

Following the current work, further research regarding the proposed probe includes: (1) quantitative evaluation of subsurface corrosion in terms of corrosion sizing involving the depth and volume; (2) assessment regarding the capability of the proposed probe in detection, sizing and shape determination of the practical/natural corrosion; and (3) signal/image processing for detection and sizing of superficial and small-volume corrosion.

## Figures and Tables

**Table 1:** Parameters of the probe.

Coil Parameter	Value
Inner length, 2*y*_0_ (mm)	24.0
Inner width, 2*z*_0_ (mm)	12.0
Height, *H* (mm)	20.3
Winding thickness, *c* (mm)	1.2
Lift-off, *z_c_* (mm)	1.0
Number of turns, *N*	289
Sensor stand-off, *z_s_* (mm)	0.5

**Table 2:** Parameters of the conductive plate.

Plate Parameter	Value
Thickness, *d* (mm)	6.0
Conductivity, *σ*_1_ (MS/m)	34.2
Relative permeability *μ*_1_	1.0
Length, *h_y_* (mm)	300
Width, *h_x_* (mm)	300

**Table 3:** Profiles and sizes of subsurface corrosion.

Corrosion Number	Corrosion Profile (in XY Plane)	Sizes
#1	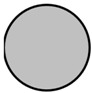	30 mm × 5 mm (diameter × depth)
#2		20 mm × 5 mm (diameter × depth)
#3		30 mm × 4 mm (diameter × depth)
#4		20 mm × 4 mm (diameter × depth)
#5	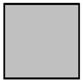	20 mm × 20 mm × 4 mm (length × width × depth)
#6	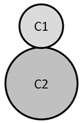	C1: 20 mm × 4 mm (diameter × depth) C2: 30 mm × 4 mm (diameter × depth)

**Table 4:** Comparison between the estimated corrosion opening size and true values.

Corrosion Number	True Diameter/Length	Estimated Diameter/Length	Relative Error
#1	30 mm	30.3 mm	1.0%
#2	20 mm	19.7 mm	1.5%
#3	30 mm	28.6 mm	4.7%
#4	20 mm	21.8 mm	9.0%
#5	20 mm	18.5 mm	7.5%
#6	C1: 20 mm	C1: 21.0 mm	C1: 5.0%
	C2: 30 mm	C2: 29.1 mm	C2: 3.0%
